# The UniProtKB guide to the human proteome

**DOI:** 10.1093/database/bav120

**Published:** 2016-02-19

**Authors:** Lionel Breuza, Sylvain Poux, Anne Estreicher, Maria Livia Famiglietti, Michele Magrane, Michael Tognolli, Alan Bridge, Delphine Baratin, Nicole Redaschi, The UniProt Consortium

**Affiliations:** 1 SIB Swiss Institute of Bioinformatics, Centre Medical Universitaire, 1 Rue Michel Servet, Geneva 4, 1211, Switzerland; 2 European Molecular Biology Laboratory, European Bioinformatics Institute (EMBL-EBI), Wellcome Trust Genome Campus, Hinxton, Cambridge, CB10 1SD, UK; 3 Protein Information Resource, University of Delaware, 15 Innovation Way, Suite 205, Newark, DE, 19711, USA; 4 Protein Information Resource, Georgetown University Medical Center, 3300 Whitehaven Street North West, Suite 1200, Washington, DC, 20007, USA

## Abstract

Advances in high-throughput and advanced technologies allow researchers to routinely perform whole genome and proteome analysis. For this purpose, they need high-quality resources providing comprehensive gene and protein sets for their organisms of interest. Using the example of the human proteome, we will describe the content of a complete proteome in the UniProt Knowledgebase (UniProtKB). We will show how manual expert curation of UniProtKB/Swiss-Prot is complemented by expert-driven automatic annotation to build a comprehensive, high-quality and traceable resource. We will also illustrate how the complexity of the human proteome is captured and structured in UniProtKB.

**Database URL**: www.uniprot.org

## Introduction

The human proteome, as we define it in UniProt, is the set of protein sequences that can be derived by translation of all protein-coding genes of the human reference genome, including alternative products such as splice variants. Although curation of human proteins has always constituted the top priority in the UniProt Knowledgebase (UniProtKB), the content of the human proteome in UniProtKB has evolved greatly in recent years, partly due to advances in technologies.

The recent rise of big data and high-throughput technologies has shifted a number of paradigms in the scientific community. Although for decades, researchers focused on a single gene and its products, it is now common to work with whole genomes and proteomes. New technologies generate large amounts of data in different fields that can be handled thanks to increases in computing and storage capacities. For instance, next-generation sequencing technologies have accelerated the identification of new genetic variants and disease-associated genes (1). Similarly, high-throughput mass spectrometry-based proteomic studies recently allowed the production of draft maps of the human proteome (2, 3) and conserved macromolecular complexes in metazoans (4). For UniProtKB, it is crucial to adapt to the challenge of the data revolution by providing the scientific community with comprehensive and non-redundant sets of proteins for each organism. It is also essential to deal with this increasing amount of data without sacrificing quality. Although quality and relevance of data continue to improve, there is still a lot of heterogeneity within and between datasets. The publications of the draft human proteome mentioned above illustrate the problem (2, 3). Differences exist between these two analyses and evaluation of these data by an independent group suggests that they should be considered with caution. Indeed, they may overestimate the number of protein-coding genes they identify (5). It is therefore important to have a set of quality filters for high-throughput data, before integrating them.

Beside high-throughput technologies, we also have to take into consideration that the requirements of our users are quite diverse. Although some users need a summary of knowledge in free text, others need structured and machine-readable information. Some users want a complete set of protein sequences derived from the reference genome, while others need expertly curated variations mapped to the reference genome or a list of variations associated with a phenotype. UniProt has to take into consideration these varied needs to build a comprehensive and high-quality resource.

UniProtKB is composed of two sections: Swiss-Prot, the reviewed section containing manually curated records with information extracted from the literature and TrEMBL, the unreviewed section with automatically annotated records. We will describe how they complement each other to offer the scientific community an expertly curated human proteome. Literature-based expert curation is essential to capture the complexity of the human proteome, but we will describe how an expert-driven automatic pipeline can complement it to filter and integrate reliable high-throughput data into UniProtKB.

## The human reference proteome in UniProtKB

When our project to manually curate all human protein-coding genes was initiated, we were first focusing on proteins that were characterized in the literature, or described in other databases, such as HGNC (6), OMIM (7) or the Human Protein Atlas (8). However, we rapidly realized that we were missing proteins encoded by genes that could only be predicted by automated genome annotation pipelines. To systematically complete the human catalogue of proteins, we therefore closely collaborated with the Ensembl group (9), and compared both UniProtKB and Ensembl human protein sets. Ensembl provided us with sequences for predicted protein-coding genes that were missing within the UniProtKB set. Using different types of evidences, including conservation, transcription, translation and characterization in the literature, we validated or invalidated these models to come up with a first complete human proteome in 2008.

All known human protein-coding genes are expertly curated and described in 20 197 UniProtKB/Swiss-Prot entries (numbers in this publication are from UniProt release 2015_10). Gene-centric versus protein-centric curation of proteomes is a matter of debate in the scientific community (10). In UniProtKB/Swiss-Prot, we have opted for a gene-centric view of the protein space. As far as possible, all protein products encoded by one gene are described in a single entry. Each entry contains a unique canonical sequence, which corresponds to the most representative sequence. In addition to these canonical sequences, 21 931 additional splice isoforms are curated, bringing the total of expertly reviewed sequences for the human proteome to 42 128. The canonical sequence as well as the sequence of splice isoforms are described in the ‘Sequences’ section of entries (see e.g. UniProtKB P15692) (http://www.uniprot.org/help/sequences_section).

**Figure 1.**
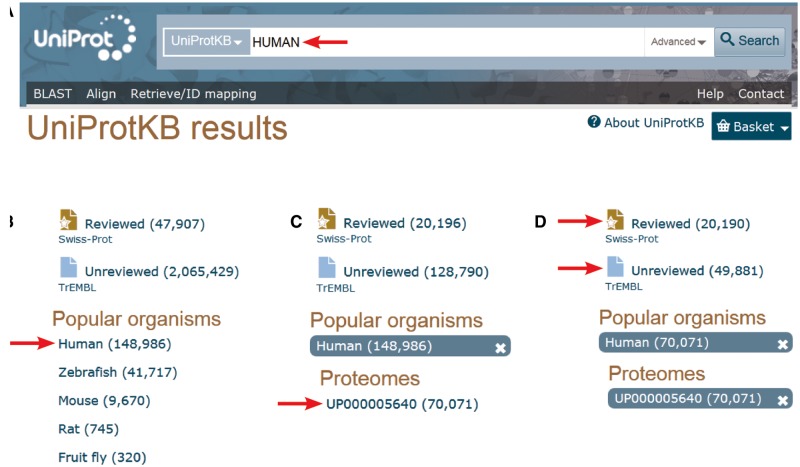
Accessing the human proteome from the UniProt web site (http://www.uniprot.org). **A.** One can directly type ‘HUMAN’ in the search box. **B.** Then select ‘Human’ in the ‘Popular organisms’ section on the left.**C.** There is a single proteome in the ‘Proteomes’ section for this organism, UP000005640, a direct link allowing access to the entries composing the human complete proteome. D. There, one still has the possibility to select the 20 197 expertly ‘Reviewed’ entries of the Swiss-Prot section of UniProtKB from the 49 496 additional ‘unreviewed’ entries of UniProtKB/TrEMBL.

With 42 128 curated sequences, it is clear that the human proteome manually annotated in UniProtKB/Swiss-Prot remains incomplete. The expert curation of all functional protein isoforms produced by alternative splicing will require more resources and time. However, a complementary pipeline for the import of predicted human protein sequences in UniProtKB/TrEMBL has been developed in collaboration with Ensembl to complete the set of human isoform sequences. >49 000 additional predicted alternative products are currently available in UniProtKB/TrEMBL. An easy way to access the human proteome annotated by the UniProt Consortium (http://www.uniprot.org/) is shown in Figure 1. Alternative ways are described in the documentation/help pages (http://www.uniprot.org/help/human_proteome).

## Sequence curation in UniProtKB/Swiss-Prot

Since the release of the first UniProtKB complete human proteome in 2008, we have continued to review and update the human proteome to reflect the evolution of knowledge and to ensure the consistency of curated sequences with the reference human genome. We re-curate sequences that are inconsistent, deprecate entries for proteins which are shown not to exist and create entries describing the products of newly identified protein-coding genes. To facilitate the usage of our resource and promote linking with other databases, curated sequences in UniProtKB/Swiss-Prot are based on the human reference genome. Although we had been curating human protein sequences for >15 years at the time of the release of the human reference genome, we have had to review thousands of sequences that differed from the reference genome. This work is done in collaboration with the HGNC, Havana (http://vega.sanger.ac.uk/index.html), Ensembl and RefSeq (11) groups and the Genome Reference Consortium (GRC) (12). We collaborate with the Consensus CDS (CCDS) project that aims to define, for each human protein-coding gene, a ‘gold-standard’ core set of protein annotations matching the translation of the reference genome (13). By comparing the UniProtKB/Swiss-Prot and CCDS protein sets, we can improve both. In most cases, discrepancies between the CCDS and UniProtKB/Swiss-Prot are due to small sequence conflicts or variations. Sometimes we have to modify the UniProtKB/Swiss-Prot sequence, whereas in other cases we request an update of the CCDS model because UniProtKB/Swiss-Prot sequences are consistent with current knowledge and evidence. Although 5 years ago, only 50% of human CCDS models had an identical counterpart in Swiss-Prot, 95% of CCDS sequences are now represented by a UniProtKB/Swiss-Prot sequence. The remaining 5% are mainly complex cases that are still under discussion or new CCDS sequences that have not been reviewed yet.

The proteome content continues to evolve as new information becomes available. The last major release of the human genome assembly was published in December 2013 (GRCh38), with new centromere representation, better segmental duplication accuracy, fewer assembly gaps and thousands of rare bases corrected (http://genomeref.blogspot.co.uk/2013http:///12/announcing-grch38.html). We continuously review and update our protein sequences to make sure that they correspond to the latest version of the genome reference assembly and send feedback to the GRC when we identify potential issues within the assemblies. For this, we directly use the ‘Report an issue’ pipeline available from the GRC website which allows us to exchange with GRC curators rapidly and efficiently (http://www.ncbi.nlm.nih.gov/projects/genome/assembly/grc/).

While we improve the quality of the reviewed protein sequences described in UniProtKB/Swiss-Prot entries, we also regularly review dubious entries describing the products of genes for which convincing supporting evidence is lacking. In UniProtKB/Swiss-Prot, the existence of 589 proteins still needs to be confirmed and these entries are currently flagged as ‘uncertain’ (for detailed information on protein existence flags see: http://www.uniprot.org/help/protein_existence). More than half of them are the predicted translation products of putative pseudogenes and their existence is a matter of debate between collaborating resources involved in gene and protein curation. When a consensus is reached on true pseudogene status, the dubious entries are deleted from UniProtKB/Swiss-Prot. This was recently the case for the CASP16 gene. After reexamination and exchanges between resources, this putative protein-coding gene was reconsidered a pseudogene by Havana, HGNC, RefSeq and UniProtKB, and the corresponding record (UniProtKB P0CB46) was deleted from release 2015_10 of UniProt. Deleted sequences are still available in UniParc (http://www.uniprot.org/uniparc/), the sequence archive of UniProt. On the other hand, the status of ‘uncertain’ proteins can be changed when evidence confirms the existence of the protein. An example is provided by the entry encoded by the NLRP2P gene (UniProtKB P0DMW2), which was considered to be a processed pseudogene until recently when it was shown to encode a functional protein (14) and its annotation was therefore updated in UniProtKB/Swiss-Prot to take into account this new information.

We also screen the literature for reports identifying new functional proteins overlooked by classical prediction and experimental methods, such as small open reading frames (smORFs). Although thousands of smORFs have the potential to encode peptides, the number of smORFs actually translated into functional proteins is difficult to predict (15). For this reason, only those supported by strong experimental evidence of expression and function are curated in UniProtKB/Swiss-Prot. A good example of a functional smORF is the myoregulin peptide, which acts as a key regulator of skeletal muscle activity (16) (UniProtKB P0DMT0). Besides smORFS, new functional proteins are regularly being discovered and characterized in the literature. This is the case for meikin (UniProtKB A0A087WXM9), which has been recently characterized in mouse and identified in human on the basis of its conservation across species (17). State of the art techniques that we use as supporting evidences of the protein-coding potential of a gene, such as ribosome profiling and other innovative methods, are constantly evolving. Ultimately, these tools may help us make a decision about the deletion of a protein sequence from UniProtKB/Swiss-Prot or on the contrary its annotation in the database. In 2014, 40 new human proteins were integrated in UniProtKB/Swiss-Prot and 117 were deprecated.

## Expert curation in UniProtKB/Swiss-Prot: increased complexity at protein level for human proteins

Literature-based expert curation is considered the gold-standard of functional annotation and constitutes a core activity of the UniProt Consortium. It is very reliable and provides high-quality annotations for experimentally characterized proteins across diverse protein families. The human proteome is certainly the best curated corpus of proteins in UniProtKB/Swiss-Prot: human proteins contain knowledge captured from >65 000 unique publications indexed in PubMed, which are curated to UniProtKB/Swiss-Prot standards. In total, if we consider knowledge inferred by similarity to mammalian model organisms such as mouse or rat, the human proteome set contains information extracted from >100 000 unique articles.

Expert curation requires in-depth reading of full-text articles before knowledge is summarized in sections dedicated to the different aspects of protein biology including function, subcellular location, or interactions with other proteins. Knowledge is represented both in a comprehensive summary that provides a complete overview of the information available, and in standardized vocabularies to facilitate subsequent retrieval whenever possible. For more information regarding expert curation, read (18, 19). Every piece of knowledge is associated with the source of the information and the type of supporting evidence, using the evidence ontology (ECO) (20).

We attach utmost importance to publications reporting events that affect the protein sequence and increase protein complexity. Most UniProtKB protein sequences come from the *in silico* translation of coding sequences (CDS) submitted to the International Nucleotide Sequence Database Collaboration. However, transcriptional and translational events can alter the genetic information and result in the production of a protein with a sequence differing from the predicted one. These events are not easily predictable and information based on experimental results, retrieved from the literature through expert curation, is the only way to reconstitute the accurate protein sequence as it is produced in cells.

RNA editing is an interesting way to introduce post-transcriptional alteration in mRNA coding sequences (21). In primates, nearly 95% of RNA editing concerns A-to-I conversion: when occurring within mRNA coding sequences, inosine is interpreted as guanosine by the translational machinery and the result may be an amino acid substitution, which can have a profound effect on protein function (21, 22). Neurotransmitter receptors, such as the glutamate receptor GRIK2 (UniProtKB Q13002), and other brain-specific transcripts are among the best characterized recoding targets for editing. C-to-U and U-to-C are also observed, as well as uracil insertion (23). In UniProtKB/Swiss-Prot, RNA editing is described in the ‘Sequences’ section (UniProtKB P07305 and P05114) and RNA edited entries can be retrieved with the keyword: ‘RNA editing’.

For a small, but growing number of mammalian mRNAs, it is known that translation initiates at a non-AUG codon. In this context, CUG, the leucine codon, is the most frequently used for non-canonical initiation. In UniProtKB/Swiss-Prot, we follow the general consensus and translate non-canonical initiator codons as methionine by default. A ‘Sequence caution’ comment in ‘Sequences’ section indicates which initiator codon is used (e.g. see UniProtKB Q8TBF5).

The presence of selenocysteine (Sec) in proteins, an amino acid similar to cysteine but that contains a selenium atom in place of sulfur, is difficult to predict. In the appropriate context, some UGA termination codons can be considered by the ribosome as a signal for the incorporation of Sec. In UniProtKB/Swiss-Prot, there are currently 26 human entries describing proteins with Sec in their sequence. These entries can be retrieved with the keyword ‘Selenocysteine’ (e.g. see UniProtKB Q9NZV5).

Once synthesized, proteins undergo numerous post-translational modifications (PTMs) that can have profound effects on their function and properties. Although hundreds of different types of PTMs are known, from the addition of simple chemical groups or complex molecules onto amino acids to proteolytic processing, a limited number can be accurately predicted. In UniProtKB/Swiss-Prot, 60% of human entries contain PTM information and >72 000 modifications of residues are expertly curated, including >5700 post-translation peptide processing events. Experimental data can be distinguished from data propagated from a related experimentally characterized protein or predicted by a program through the use of ECO codes as discussed above. PTMs are described using a controlled vocabulary: a list of all terms used in UniProtKB is available in the ptmlist.txt document (http://www.uniprot.org/docs/ptmlist).

Capturing post-transcriptional and post-translational modifications from the literature is essential to reflect the complexity of the human proteome. Over the years, UniProtKB/Swiss-Prot has catalogued these events in a comprehensive manner, and these data can be used both as a high-quality training set for development and enhancement of bioinformatics algorithms, and as an essential library for the identification of proteins by proteomics.

## Human variations in UniProtKB/Swiss-Prot

Although it is essential to describe a reference human proteome to which we curate functional knowledge, it is equally important to capture the diversity of that proteome within the human population. Variability is high between the genomes of two unrelated individuals with an estimated difference every one thousand base pairs, and a total of ∼3.3 million single nucleotide polymorphisms (SNPs). Although the majority of these SNPs are neutral, i.e. not altering the function of the protein, some dramatically alter proteins and are responsible for phenotypes and diseases (24).

In UniProtKB/Swiss-Prot, priority is given to the curation of single amino acid polymorphisms associated with diseases and phenotypes described in peer-reviewed literature. Currently, 72 960 genetic variants are annotated in UniProtKB/Swiss-Prot entries. 40% of them are associated with a genetic disease and 12% contain information concerning their functional consequence on proteins. This indicates that a large fraction of variants are of unknown significance with respect to protein function and that our biochemical and cellular knowledge is still scarce. All UniProtKB/Swiss-Prot variants can be found in the humsavar.txt table (http://www.uniprot.org/docs/humsavar) and for each protein, in the ‘Sequences’ section (http://www.uniprot.org/help/sequences_section).

**Figure 2.**
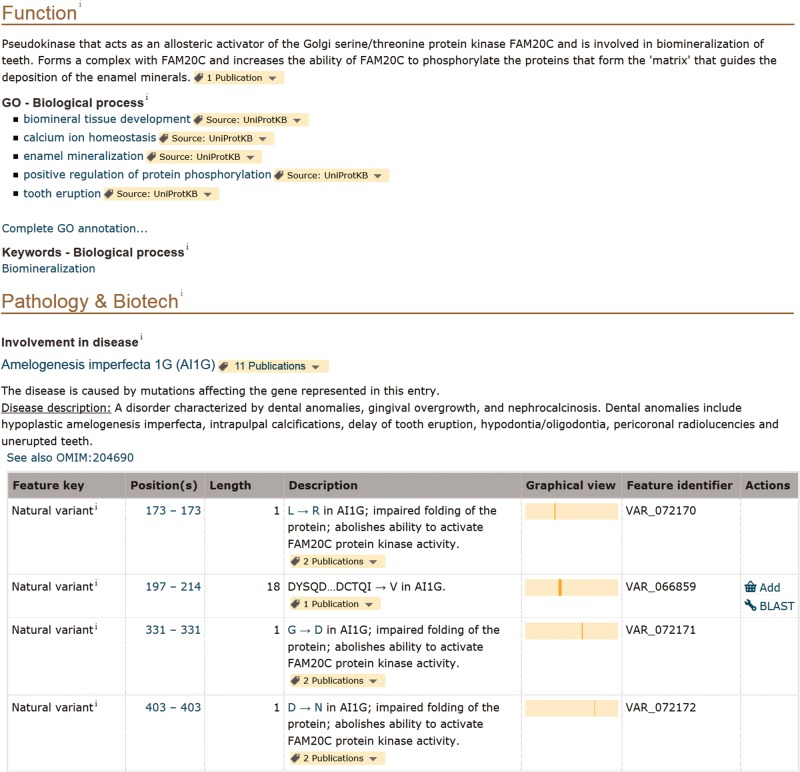
Screenshot of the ‘Function’ and ‘Pathology and Biotech’ sections of human FAM20A entry (UniProtKB Q96MK3, http://www.uniprot.org/uniprot/Q96MK3).

Information related to disease is found in the ‘Pathology and Biotech’ section of entries (http://www.uniprot.org/help/pathology_and_biotech_section). For instance, in this section of the entry describing the pseudokinase FAM20A (UniProtKB Q96MK3), we mention that the gene is associated with a form of amelogenesis imperfecta, a disorder affecting the dental enamel (Figure 2). The disease is described in a structured format based on OMIM disease nomenclature, if available, and contains cross-references to the OMIM database and MeSH terms [detailed information about human variations and diseases in UniProtKB/Swiss-Prot is described in (25)]. Strong added value comes from expert curation of the detailed molecular characterization of protein variants. We specifically capture their effect on protein properties in terms of function, localization, interaction and PTM among others. We annotate these effects taking into account what is already described in the whole entry, principally in the ‘Function’, ‘Subcellular location’, ‘Interaction’ or ‘PTM/Processing’ sections. For FAM20A, there are six natural variants annotated in the entry. Four of them, associated with amelogenesis imperfecta, are reported in the ‘Pathology and Biotech’ section together with their characterization when available. They affect the ability of FAM20A to activate the Golgi serine/threonine protein kinase FAM20C, which is described in the ‘Function’ section of the entry (Figure 2). One can therefore clearly make a link between the pathology and the function of the protein. Free text description of variants that are functionally characterized is however not readable by a computer and we are working on improving their representation. In order to facilitate search and retrieval of these variants, free text annotations will be restructured using a combination of controlled vocabularies to describe variant effects (*Famiglietti et al.*, in preparation).

Recent advances in sequencing technology generate large datasets of variants that provide a comprehensive view of human genetic variation (1). It is therefore important to complement the ongoing expert curation of variants from the literature with the incorporation of variants from large-scale projects into UniProtKB. Variants from the 1000 Genomes Project and COSMIC release v71 are automatically mapped to UniProtKB and can be found on the UniProt FTP server, in the homo_sapiens_variation.txt.gz file that contains a catalogue of novel SNPs for both UniProtKB/Swiss-Prot and UniProtKB/TrEMBL sequences (http://ftp://ftp.uniprot.org/pub/databases/uniprot/current_release/knowledgebase/variants/).

## Use of proteomics data to confirm human proteome complexity

Results from mass spectrometry-based proteomics experiments also constitute a valuable source of information for the curation of the human proteome. We use them to confirm the existence of proteins and protein PTMs. However, a number of challenges exist for integrating high-throughput proteomics data in UniProtKB. Publications and dataset reports from proteomics experiments exhibit highly variable levels of quality and reliability. This is due to the heterogeneity of proteomics experimental protocols on one side, and to the computational and interpretational stringency of results on the other side. Because the overall number of false identifications increases with the number of incorporated datasets, the integration of peptide identifications that can tolerate up to 1% of false positives may lead to the accumulation and propagation of erroneous annotations. This might be of potentially high cost in time and resources if a false positives protein identification is at the origin of further biological studies and it can undermine the value of databases such as UniProtKB (26).

**Figure 3.**
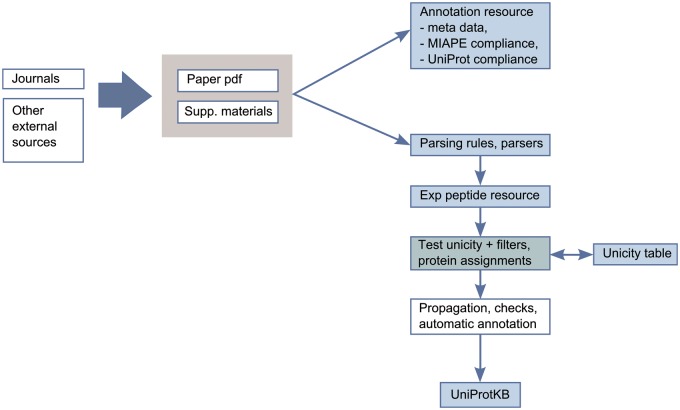
Annotation scheme for integration of proteomics data in UniProtKB.

To limit the number of false positives and only integrate reliable data, we have developed an expert-driven analysis pipeline for integration of proteomics data into UniProtKB/Swiss-Prot (Figure 3). The pipeline consists of the evaluation of publications by curators with expertise in proteomics. We first confirm that the publications are compliant with the MIAPE (Minimum Information About a Proteomics Experiment) standard for reporting proteomics experiments, providing access to the raw data and the associated metadata (27). The relevance of the scientific articles and the methods used, such as the precision of the instruments, the peptide identification software used, the selection cut-off values and the post-processing methods are also reviewed.

After this first evaluation step, we completely reprocess the identified peptide data from publications. Extracting data from publications can be challenging since results are provided in heterogeneous formats (such as PDF files, Excel spreadsheets, Supplementary Materials and links to authors webpages). We implemented a robust extraction and filtering system to keep only reliable and consistent information. Mass spectrometry data are not re-analysed but stringent cut-offs are applied on the peptide scores provided in the publications to remove dubious identifications (28, 29). We also check the biological relevance of the PTMs (a phosphorylation site should not be located in a transmembrane region, e.g.). Ultimately, the identification of the protein has to be unambiguous. Most of the identification software uses a parsimony approach, i.e. a minimal list of proteins that may produce the identified peptides is used. This does not mean that the reported protein is definitely in the sample or that other proteins are not present and generally, this does not allow unambiguous assignment of a peptide to a protein. To address these issues, each peptide is compared to a ‘Unicity’ table, which contains all theoretical unique peptides from the complete human proteome. This table also takes into account experimental and predicted sequence annotations from UniProtKB/Swiss-Prot, including isoform differences, sequence processing events and natural variants.

For example, Bian *et al.* (30) recently analysed the liver phosphoproteome and reported the identification of 55 061 peptides for 22 446 phosphorylation sites in 6526 different proteins. After reprocessing and assessment of their results according to stringent filtering rules [such as a minimum Mascot score of 40 or a posterior error probability (PEP) of (lt)1% or a minimum PTM localization score (Ascore) of 19], only 26 497 unique peptides are validated, and 5197 phosphorylation sites are annotated in 4118 UniProtKB/Swiss-Prot entries (28, 29, 31).

Such stringent rules allow limiting heterogeneity between different publications, reducing the number of false positives to only extract gold-standard knowledge. So far, we have evaluated 65 high-throughput proteomics articles dealing with human samples. 39 were integrated into UniProtKB/Swiss-Prot while 26 were not considered because the methodology did not match our quality criteria or because the data were not fully accessible. From these 39 publications, 90 889 peptides passed the filtering steps, enriching the human proteome of 23 092 PTMs through 5822 UniProtKB/Swiss-Prot entries.

In addition to the above approach which provides high-quality proteomics data from published literature, we have also developed an automated pipeline to provide mappings of identified human peptides from public mass spectrometry proteomics repositories to UniProtKB sequences. These are available in a dedicated ‘proteomics_mapping’ directory on the UniProt FTP site (http://ftp://ftp.uniprot.org/pub/databases/uniprot/current_release/knowledgebase/proteomics_mapping/) together with a description of how the mappings are generated. This includes peptides from PeptideAtlas (32) and MaxQB (33) for the human proteome as well as for a number of other species. In future UniProt releases, we plan to add data from more repositories. To ensure data quality, the identifications are filtered based on thresholds set by us according to the global quality metrics provided by each proteomics resource. These peptide mappings greatly increase the proportion of human proteins in UniProtKB whose existence is supported by experimental proteomics data with the pipeline providing mass spectrometry evidence for 69 639 human proteome sequences.

## Discussion

Curation of the human proteome remains challenging in the era of big data. Manual expert curation is a costly and time-consuming process. Should it then be considered as outdated and useless in view of the amount of data to be dealt with? In UniProt, we believe quite the opposite and regard it as the cornerstone of knowledgebases. We think expert curation remains essential for two reasons. First, because the majority of publications still describe in depth characterization of gene products, with complex knowledge that cannot be captured by machine learning or text mining technologies. For such publications, expert curation is by far the most reliable and robust method to report information. Second, because high-throughput studies generate a higher rate of false positives than classical assays, and cannot be integrated without safeguards.

To retain its value, expert curation should however be able to cope with the increasing amount of data. With >500 000 records indexed yearly in PubMed, a careful prioritization of both articles and proteins to curate is necessary. Publications of high quality, that provide a detailed picture of protein function and characteristics should be curated as a priority while others might not be considered. Scientific literature is extremely redundant: for instance, of 285 indexed in PubMed only 15 have been used for the recent re-curation of the CYP4F2 enzyme (UniProtKB P78329). Other publications are either not relevant for curation or present redundant and/or weaker data. Collaborations with other resources are also crucial, because they ensure data consistency, facilitate exchanges, prevent duplication of the curation effort and thereby enhance the efficiency of expert curation. Our collaborations with the HGNC, Havana, Ensembl and RefSeq groups and the GRC on sequence curation in human constitutes an excellent example of a mutually beneficial collaboration and, most importantly, beneficial to the community. In this context, we participated with other annotation resources in defining guidelines for the validation of novel human protein coding loci in a workshop held at the Wellcome Genome Campus in May 2015 (34).

Moreover, expert curation should be in constant evolution to ensure that it meets user expectations. In UniProtKB/Swiss-Prot, we have considerably improved traceability and representation of knowledge to enhance the usability of the resource. We have also restructured a number of annotations into a machine-readable format in order to improve access to this knowledge. We recently modified the format of the ‘Cofactor’ subsection and introduced a controlled vocabulary from ChEBI to describe the cofactors (35). We are now working on the representation of variants associated with functional characterization (*Famiglietti et al.*, 2014). In the future, we plan to restructure other annotation fields such as the ‘catalytic activity’ comment in collaboration with Rhea (36).

It is quite clear that high-throughput experiments cannot be ignored and that many studies provide valuable information which is extremely useful for a number of users. Moreover, efforts in the proteomics community in recent years have improved the downstream integration of data, and coordination through the proteomeXchange has shown benefits in the selection of relevant datasets (37). However, such studies should be reviewed by expert curators with predefined stringent quality criteria and the origin of data from these studies should be clearly visible for users. The expert-driven pipeline for integration of proteomics data, which contains a mixture of manual and automatic procedures, constitutes a good illustration of integration of high-throughput studies without sacrificing quality. Before integration, each article is reviewed by a curator and a number of filtering steps are applied in order to limit the number of false positives. It prevents for instance, over-annotation of similar proteins like paralogs and putative products of pseudogenes. This very stringent approach has some limitations by excluding potentially valid identifications. However, it is necessary to ensure that only gold-standard information is added to reviewed entries. This pipeline is also applicable to other organisms. A number of publications describing proteomics data in other reference organisms such as *Mus* *musculus*, *Rattus* *norvegicus*, *Arabidopsis* *thaliana*, *Saccharomyces* *cerevisiae* and *Mycobacterium* *tuberculosis* have been curated. In total, from the 77 curated publications, we extracted and verified around 234 000 unique peptides covering 26 236 UniProtKB/Swiss-Prot entries and generating the annotation of 53 519 different PTMs.

Traceability of information is also essential to inform users about the reliability of information, particularly when resources contain a combination of expert and automatic annotations. We always separate expert curation from automatically generated information in UniProt: all protein-coding genes and well-characterized alternative splicing sequences of the human proteome are stored in UniProtKB/Swiss-Prot, while unreviewed sequences corresponding to predicted alternative splicing sequences are stored in UniProtKB/TrEMBL. They complement each other to build a complete proteome. We apply the same principle to protein variants: variants described and confirmed in the literature which have been reviewed by UniProt curators can be found in UniProtKB/Swiss-Prot and in the humsavar.txt table**(**http://www.uniprot.org/docs/humsavar), while variants automatically imported from other resources are available in a distinct file on the UniProt FTP site. Similarly, data from published high-throughput proteomics studies are labeled in UniProtKB/Swiss-Prot with a distinct ECO evidence code (ECO code ECO:0000244) to inform users that such knowledge is derived from a combination of experimental and computational analysis) while automatically generated mappings to peptides in proteomics repositories are available on the UniProt FTP site.

The human proteome is prepared by the UniProt Consortium through a process of expert curation that continuously evolves to adapt to changing knowledge, new technologies and the changing needs of its users. The high complexity of the human proteome results from the combination of post-transcriptional and post-translational modifications. Capturing the complexity of the protein world is what we try to reflect in UniProtKB and constitutes a major challenge for proteome curation. From this perspective, the human proteome paves the way for the curation of other proteomes.
